# Evaluation of the Effectiveness of a Mouthwash Containing Spilanthol and Cannabidiol on Improving Oral Health in Patients with Gingivitis—Clinical Trial

**DOI:** 10.3390/jcm14051641

**Published:** 2025-02-28

**Authors:** Maksymilian Kiełbratowski, Anna Kuśka-Kiełbratowska, Anna Mertas, Elżbieta Bobela, Rafał Wiench, Małgorzata Kępa, Agata Trzcionka, Rafał Korkosz, Marta Tanasiewicz

**Affiliations:** 1 Department of Conservative Dentistry with Endodontics, Faculty of Medical Sciences in Zabrze, Medical University of Silesia, 40-055 Katowice, Poland; mkielbratowski@sum.edu.pl (M.K.); atrzcionka@sum.edu.pl (A.T.); martatanasiewicz@sum.edu.pl (M.T.); 2 Department of Periodontal Diseases and Oral Mucosa Diseases, Faculty of Medical Sciences in Zabrze, Medical University of Silesia, 40-055 Katowice, Poland; rwiench@sum.edu.pl; 3 Department of Microbiology and Immunology, Faculty of Medical Sciences in Zabrze, Medical University of Silesia, 40-055 Katowice, Poland; amertas@sum.edu.pl (A.M.); ebobela@sum.edu.pl (E.B.); 4 Department of Microbiology, Faculty of Pharmaceutical Sciences in Sosnowiec, Medical University of Silesia in Katowice, 41-200 Sosnowiec, Poland; mkepa@sum.edu.pl

**Keywords:** spilanthol, tea tree oil, mouthwashes

## Abstract

**Background/Objectives**: Plaque-associated gingivitis is widely regarded as a local inflammatory condition initiated by the accumulation of a non-specific dental biofilm in the interaction with the host immune system. The initial symptom noticed by the patient is bleeding gums. The use of mouthwash can serve to supplement mechanotherapy. However, there is an increasing interest in mouthwashes comprising natural ingredients, including cannabidiol (CBD) and spilanthol. The objective of this study was to evaluate the effect of an oral rinse containing spilanthol and CBD oil compared to a rinse containing tea tree oil on the oral microbiota and the values of selected oral status indicators in patients with gingivitis. **Methods**: The study included 40 patients treated with a rinse containing tea tree oil (TTO)/TTO + spilanthol + CBD for a period of 42 days. Patients rinsed their mouth twice daily for 30 s. The patients’ oral microbiome was assessed before and after treatment, and bleeding on probing (BOP) and approximal plaque index (API) were assessed. The study was double-blind. **Results**: API and BOP were reduced in all groups, both the test and control. The most significant decrease in baseline BOP-1 scores was observed in test groups A and D (*p* = 0.005062 and *p* = 0.005062, respectively). A significant difference in API improvement was observed between the initial and final visits in the test (A, D) and control (B, C) groups (*p* = 0.012516, *p* = 0.005062, *p* = 0.004028, *p* = 0.003172, respectively). **Conclusions**: Firstly, the use of a mouthwash containing cannabidiol (CBD) and spilanthol was demonstrated to be efficacious in the maintenance of oral microbiota homeostasis. Secondly, the combination of TTO with spilanthol and CBD in the rinse was shown to result in a more significant reduction in selected oral health parameters (BOP and API) and anti-inflammatory effects when compared to a rinse with TTO alone. It should be noted that this is a pilot study and will continue.

## 1. Introduction

In accordance with the most recent Classification of Periodontal and Peri-Implant Diseases and Conditions (2017), gingival diseases can be categorised as follows: firstly, gingivitis, which is characterised by the accumulation of plaque biofilm (gingivitis); and secondly, gingival diseases not attributable to plaque accumulation. The latter category will not be discussed in this publication [1]. Plaque-associated gingivitis is defined as a local inflammatory condition initiated by accumulating a non-specific dental biofilm in the interaction with the host immune system [1]. Gingival bleeding is the primary indicator of gingivitis, and the bleeding on probing indicator (BOP) has been established as the principal means of the objective, accurate, and reliable identification of gingival health status [2,3]. The distinguishing characteristic of this condition is the reversibility of the inflammation. Gingivitis is widely regarded as a pivotal precursor to the progression of periodontitis and the subsequent occurrence of progressive clinical attachment loss (CAL) around teeth. Consequently, the primary preventive strategy for periodontitis is the implementation of measures to address gingivitis at its earliest stages [3]. The early diagnosis and prevention of this condition are of paramount importance for patients exhibiting no characteristic signs of inflammation [1,4]. However, there is an awareness of a tendency to lose clinical attachment and bone at sites of gingivitis, which may depend on the susceptibility and response of the individual to the inflammatory agent [5]. The investigation of the epidemiology of gingivitis is challenging due to the existence of numerous heterogeneous studies in which different parameters were assessed. One such example is the Community Periodontal Index of Treatment Needs (CPITN). A study conducted in the United States of America in 1999 on a group of patients aged between 30 and 90 years, in which the BOP index was also assessed, showed a prevalence of gingivitis in 32.3 per cent of the subjects [6]. A study conducted in Romania (2017) on a group of adolescents aged between 10 and 17 years is worthy of note. In these studies, the GI gingival index was utilised to assess the prevalence of gingivitis, which was observed to be as high as 91% of the subjects [7]. A large-scale epidemiological study conducted on a group of individuals aged 35–44 years in Poland revealed that only 1.1% of the study participants assessed did not have symptoms recorded in the CPI. These findings underscore the prevalence of the disease and emphasise the need for comprehensive primary and secondary prevention measures that are both effective and accessible [8]. The prevention of gingivitis and its treatment according to the 2017 diagnostic management algorithms and treatment guidelines should follow the protocol outlined in Step 1 [9,10]. It is imperative that patients are made aware of the problem and motivated to perform effective oral hygiene. In addition to mechanical plaque control, the use of a mouthwash that chemically reduces biofilm build-up is recommended [11]. However, the employment of chemical agents in isolation, without the incorporation of mechanotherapy, has been shown to lack effectiveness in the control of bacterial plaque accumulation [12,13]. Scientific reports have indicated that the combination of mechanotherapy and chemical agents can yield positive outcomes. However, the selection of these agents must be tailored to the individual patient’s needs. These agents can be administered in the form of pastes, gels, or mouthwashes [14,15]. In the case of rinses, chlorhexidine digluconate is the most common choice, but it should only be used for a limited period of time due to its side effects, which include the occurrence of taste disorders and mucosal irritation [16,17]. A plethora of substances, including but not limited to cetylpyridinium chloride, triclosan, octenidine, nanosilver, and essential oils (e.g., methyl salicylate, thymol, eucalyptol, menthol), are employed in mechanotherapy support. Nevertheless, the efficacy of these substances is also limited. In view of the considerable challenge posed by the accumulation of dental biofilms and the high prevalence of gingivitis, there is a rational basis for the exploration of the identification of novel substances capable of eradicating dental biofilms while exerting minimal adverse effects on the patient’s organism [14,15,18,19]. Examples of such substances include, but are not limited to, essential oils, such as tea tree oil, as well as other substances such as spilanthol or cannabidiol. The utilisation of plant essential oils is predominantly associated with aromatherapy, a practice that has been demonstrated to elicit a marked effect of relaxation, in addition to their recognised antiseptic, analgesic, and anti-inflammatory properties. Increasingly, these oils are being used in medical and dental contexts, where their antimicrobial properties are particularly valued. The basis of their antiseptic properties is their lipophilic effect, which enables them to bind to the cell membranes of microorganisms, leading to the efflux of intracellular components and loss of cell integrity [20,21]. Another substance of note is spilanthol extract from Acmella oleracea, which belongs to the Asteraceae plant family. Phytochemical studies of the plant have identified the presence of various chemical compounds, including trans-isoferulic acid, alkylamides, 3-lipoic acid, P-sitostenone, scopoletins, and vanillic acid, as well as trans-ferulic acid. These compounds have been shown to possess a diverse range of biological activities, including anti-inflammatory, analgesic, antiallergenic, and antimicrobial properties. The latter effect is particularly notable against Gram-positive and Gram-negative bacteria, as well as *Candida* [22,23]. Cannabidiol (CBD), a non-psychotropic constituent of Cannabis sativa, has attracted considerable scientific attention due to its extensive therapeutic potential. The available evidence suggests that CBD exerts anti-inflammatory, analgesic, antioxidant, neuroprotective, and antimicrobial effects, underscoring its potential as a multifaceted therapeutic agent. The substance can be used as an adjunct to maintain oral hygiene and as an anti-inflammatory agent. This underscores the necessity for further research, including, but not limited to, clinical studies investigating the efficacy of cannabidiol in combination with other substances for the treatment of caries, gingivitis, and other oral diseases [24,25,26].

The objective of this study was to evaluate the impact of an oral rinse comprising spilanthol and CBD oil in comparison to a rinse consisting of tea tree oil on the oral microbiota and the values of specific oral status indicators in patients diagnosed with gingivitis.

## 2. Materials and Methods

### 2.1. Consent of the Bioethics Committee

The study was conducted in accordance with the ethical standards outlined in the Helsinki Declaration and was approved by the Bioethics Committee of the Silesian Medical Chamber in Katowice (no. 12/2023 of 29 May 2023). Prior to participation in the study, all participants provided written informed consent. Furthermore, each participant was informed in writing of their right to withdraw at any time.

### 2.2. Materials Used in the Study

The study utilised two mouthwashes: the first contained 0.0003% spilanthol and 1.6610% ethanol extract of CBD (designated as rinse number 1), and the second comprised essential oils, including tea tree oil at 0.105% (designated as rinse number 2).

Patients in test groups A and D were administered rinse number 1, while patients in control groups B and C were administered rinse number 2. All patients in the study and control groups were instructed to use a placebo toothpaste, which was formulated with an abrasive toothpaste base but lacked active substances. This approach was adopted to standardise the study conditions and eliminate the influence of substances derived from commercial products. The following ingredients were used in the formulation of the placebo toothpaste: water, glycerine, xanthan, gellan gum, carboxymethylcellulose, silica, sorbitol, titanium dioxide, and sodium fluoride at 1450 ppm (parts per million). The same production company, Melaleuca Poland Sp. z o.o. (Gliwice, Poland), was responsible for the formulation and production of the mouthwash and toothpaste.

### 2.3. Studied Groups of Patients

**Figure 1:**
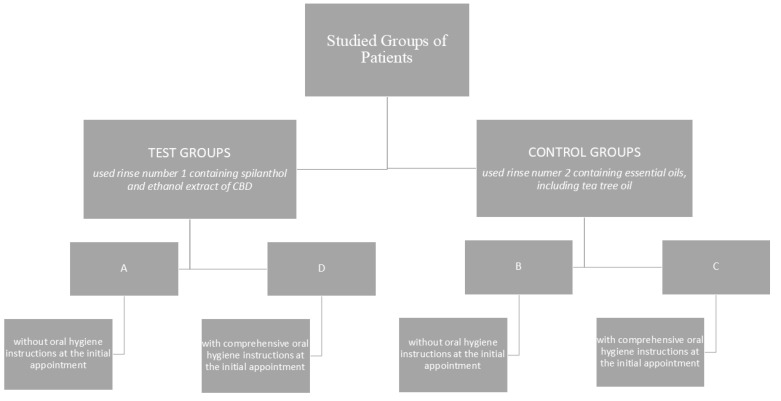
Studied groups of patients.

The test cohort comprised 40 patients diagnosed with gingivitis, aged between 18 and 45 years (mean age 31.95 ± 6.33), including 26 women and 14 men. Patients were randomly assigned to one of four groups: two test groups (A and D) and two control groups (B and C). Group A (the test group) comprised 10 patients, including five females and five males, aged between 18 and 45 years (mean age 31.4 ± 4.7 years), with BOP values of ≥10%. The patients in test group (A) were instructed to use oral rinse number 1. No detailed oral hygiene instructions were provided to this cohort of patients during the initial visit. Group B (control group) comprised 10 patients, including eight women and two men, aged between 18 and 45 years (mean age 28 ± 6.88), with BOP values ≥ 10%. The patients in this cohort were instructed to use oral rinse number two, which is commercially available, and no detailed oral hygiene instructions were provided during the initial visit. Group C, the control group, consisted of 10 patients, comprising eight females and two males aged between 18 and 45 years (mean age 36.1 ± 5.69 years) with a BOP value of ≥10%. The patients in this group used oral rinse number two. The patients in this group received comprehensive oral hygiene instructions at the initial visit. Group D (the test group) comprised 10 patients, five women and five men, aged between 18 and 45 years (mean age 32.3 ± 5.89 years), with a BOP value of ≥10%. The patients in the test group (D) were instructed to use oral rinse number 1. A comprehensive oral hygiene tutorial was provided to this cohort of patients during their initial visit, with the same investigator delivering the instructions to each patient. After obtaining the patients’ consent to participate in the study, general medical and dental interviews were conducted, followed by a detailed dental examination (Figure 1). The dental examination, in each patient, was carried out by the same investigator. Patients were given a number 1 or number 2 mouthwash and toothpaste (placebo) enough to use for 42 days. The patients were instructed to brush their teeth twice a day with the toothpaste provided. Patients who received detailed hygiene instructions were advised to carry out their brushing as prescribed, while patients who did not receive hygiene training were to do so as before. All patients were advised to use mouthwash twice a day for 30 s and to refrain from using any other oral care products until the end of the experiment. In groups C and D, oral hygiene instructions were provided. The delivery of these instructions was a combination of verbal and visual methods, with the utilisation of a model of the oral cavity employed to facilitate the comprehension of the subject matter. The roll brushing technique (rotating and sweeping) was presented as a method of achieving optimal oral hygiene, with the duration of the brushing process instructed to be two minutes. Additionally, a demonstration was conducted to illustrate the technique of cleaning interdental spaces using dental floss. The clinical trial that was conducted was of a double-blind nature.

### 2.4. Inclusion and Exclusion Criteria

The inclusion criteria were as follows: participants must have been aged between 18 and 45 years, provided written consent to participate in the study, exhibited gingival inflammation with a BOP of at least 10%, and had a minimum of two teeth present in each quadrant. The exclusion criteria were as follows: a lack of written consent to participate, the presence of periodontal disease, cancer, psychosomatic disorders, facial trauma, asthma, atopic dermatitis, food and drug allergies, or other allergic conditions, pregnancy or breastfeeding, smoking, alcohol abuse, and the use of antibiotic or antifungal drugs within the previous six months.

### 2.5. Dental Examination and Dental Indicators

**Figure 2:**
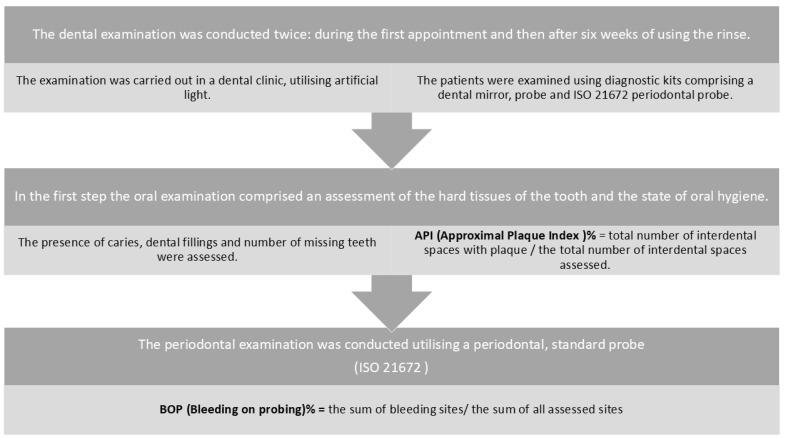
The following diagram illustrates the dental examination that was conducted at the initial visit, as well as the examination conducted after six weeks of utilising the rinse.

The dental examination was conducted in two phases: initially, at the first visit, and subsequently, after a six-week period of application. The examination was carried out in a dental clinic, utilising artificial light. The patients were examined using diagnostic kits comprising a dental mirror, probe, and periodontal probe. The latter is characterised by a 0.5 mm diameter tip, cylindrical spike design, and 1.75-degree taper. The oral examination included an assessment of the hard tissues of the tooth, such as the presence of caries, dental fillings, and the number of missing teeth, as well as an evaluation of indices. The assessment included the following indices: API and BOP. The state of oral hygiene was evaluated using the Approximal Plaque Index (API) according to the methodology proposed by Lange [27]. The index is employed for the assessment of oral hygiene in interdental spaces, which are areas of the most frequent accumulation of untreated plaque. The index’s dichotomous nature allows for the elimination of any subjective factors on the part of the researcher. The index was subjected to a series of tests, with the presence of plaque evaluated in quadrants I and III on the oral cavity side, in the interdental spaces, and in quadrants II and IV on the oral vestibule side, also in the interdental spaces. The presence of plaque is indicated by a plus sign (+), while its absence is indicated by a minus sign (−). Subsequently, a quotient was calculated, representing the total number of interdental spaces with plaque and the total number of interdental spaces assessed. The resulting value was expressed as a percentage. The level of oral hygiene was determined by the value of the index obtained. A score of 100% indicates a poor level of hygiene, 70% to 40% indicates an average level of hygiene, 39% to 25% indicates a fairly good level of hygiene, and a score below 25% indicates an optimal level of hygiene [28]. The degree of inflammation was evaluated using the ISO 21672 periodontal probe, with a particular focus on the bleeding on probing (BOP) index, as outlined by Ainamo and Baya. The assessment was conducted by probing the gingival sulcus/periodontal pocket with a standardised periodontal probe, applying a constant force of 0.25 N to the bottom of the pocket [29]. This procedure was performed at six measurement sites (buccal-mesial, buccal, buccal-distal, lingual-mesial, lingual, and lingual-distal) on all teeth present. As with the entire examination, the assessment was conducted by the same examiner who had previously been trained in the technique of applying repetitive pressure to the examination. The values were then assessed in a dichotomous yes/no manner, with the final value given as a percentage. According to the current Classification of Periodontal and Peri-Implant Diseases and Conditions (2017), a value where the number of bleeding areas with pocket depths not exceeding 3 mm was more than or equal to 10 per cent was taken as inflammation [1,30]. The index was calculated using the following formula: BOP is defined as the sum of bleeding sites divided by the total number of sites tested, expressed as a percentage (Figure 2).

### 2.6. Microbiological Examination

In each group of patients, a swab was taken from the mucosa of the floor of the mouth in a non-invasive manner. The procedure was conducted in the morning, with patients having fasted and abstained from oral hygiene prior to the procedure. The sample was taken at the initial and concluding visits, occurring approximately six weeks apart. The material for microbiological examination was collected using sterile swabs in tubes containing AMIES transport medium with carbon (DELTALAB, Rubi, Spain). Subsequently, the material was conveyed to a duly accredited laboratory facility within a 24 h period and stored at room temperature. The swabs from the floor of the mouth were inoculated onto Columbia agar with 5% sheep blood (bioMerieux SA, Marcy-l’Étoile, France) and Schaedler agar with 5% sheep blood (bioMerieux SA, Marcy-l’Étoile, France) in order to cultivate aerobic and anaerobic flora, respectively.

The inoculated media were incubated in a hothouse at 37 °C under aerobic conditions for a period of 24 h. Following incubation, colonies were aseptically collected from Columbia agar with 5% sheep blood and identified using a VITEK^®^ MS PRIME (bioMerieux SA, Marcy-l’Étoile, France). The bacteria were identified by MALDI-TOF MS (matrix-assisted laser desorption/ionisation time-of-flight mass spectrometry) technology. The process of identifying microorganisms is based on the analysis of their protein profile, whereby complex mixtures of proteins are examined using mass spectrometry. Test colonies were plated onto a VITEK MS-DS plate (smear of colonies within the marked area on the plate) and 1 µL of each of the VITEK^®^ MS CHCA matrix solutions (i.e., α-cyano-4-hydroxy cinnamic acid and acetonitrile, Bio-Rad Laboratories, Marcy-l’Étoile, France) was added. The proteins present in the test sample were attached to the matrix. Following thorough drying, the plate was inserted into an apparatus where spectra were automatically acquired. The process duration is approximately one minute. In the device, the native proteins are ionised and the time-of-flight (TOF) is measured. The identification algorithm is based on a comparison of the data to a database, which is then processed by an algorithm to obtain microbial identification.

The resulting identification results are displayed in the VITEK^®^ MS 1.1.2.36 software (bioMerieux SA, Marcy-l’Étoile, France). Microorganisms were seeded on Schaedler agar with 5% sheep blood and incubated under anaerobic conditions in a Whitley A35 anaerobic workstation microbial culture chamber (BENTLEY, Warsaw, Poland) for seven days at 37 °C. The Whitley A35 workstation provides optimal conditions for the processing, incubation, and testing of samples without exposure to atmospheric oxygen. Anaerobic conditions are maintained by a mixture consisting of 10% hydrogen, 10% carbon dioxide, and 10% nitrogen. Traces of oxygen combine with the hydrogen in the presence of a catalyst to form water vapour, which is removed to the outside. Following growth on Schaedler agar with 5% sheep blood (which allows for the growth of anaerobic bacteria for a longer period than aerobic culture), identification was conducted in a manner consistent with that employed for Columbia agar blood medium.

### 2.7. Statistical Analysis

The first stage of the statistical analysis was to check the normality of the data distribution using the Shapiro–Wilk test. Levene’s test was then used to check for homogeneity of variances. The results of the indicator values were presented as arithmetic means. The NIR test was used to make comparisons between groups. The test group was contrasted with the control group for API and BOP using Student’s *t*-test for dependent samples. Statistically significant differences in the parameters assessed were considered to have occurred when *p* < 0.05.

## 3. Results

### 3.1. Bleeding on Probing (BOP)

**Figure 3:**
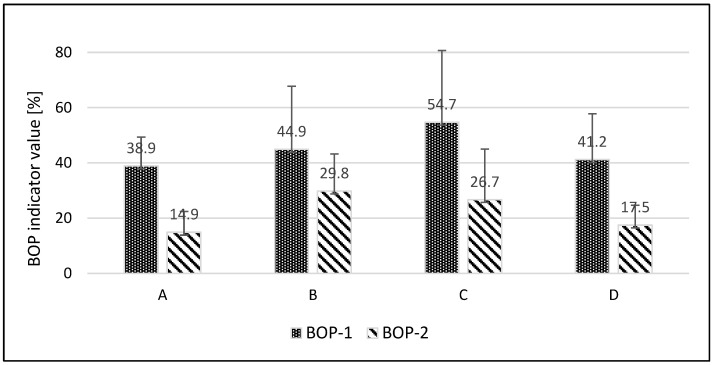
The mean percentage with standard deviation of the BOP index in the test groups (A and D) and control groups (B and C) is presented herewith. BOP-1: value assessed during the first visit. BOP-2: value assessed at the follow-up visit after 6 weeks of study.

In order to be eligible for inclusion in the study, patients were required to have a baseline BOP of 10% or greater, across all four groups (A–D). Patients with a baseline BOP between 10 and 100%, indicating the presence of gingival inflammation, were randomly allocated to either the study or control groups. No statistically significant differences were observed in the BOP-1 (BOP-1: value assessed during the first visit) variable between the four groups (A vs. B *p* = 0.504335, A vs. C *p* = 0.084178, A vs. D *p* = 0.797464, B vs. C *p* = 0.277947, B vs. D *p* = 0.679945, C vs. D *p* = 0.137872). For the BOP-2 (BOP-2: value assessed at follow-up visit after 6 weeks of study) variable, statistical significance was observed for the following comparisons: groups A and B, A and C, and B and D (*p* = 0.01144, *p* = 0.041799, *p* = 0.034289, respectively). It is worthy to note that patients in the aforementioned comparison groups utilised disparate rinses. No statistically significant difference was observed between groups A and D, B and C, or C and D. The largest reduction in baseline BOP-1 scores was noted for test groups A and D, and in both groups, the difference between these scores was statistically significant (*p* = 0.005062 and *p* = 0.005062, respectively) (Figure 3). Furthermore, a statistically significant difference was observed between the BOP-1 and BOP-2 values for the control group (*p* = 0.012516). The results for the control group B did not reach a statistically significant level. Furthermore, a markedly statistically significant discrepancy in BOP (BOP-1 vs. BOP-2) was substantiated for patients across all groups (*p* = 0.005848).

**Figure 4:**
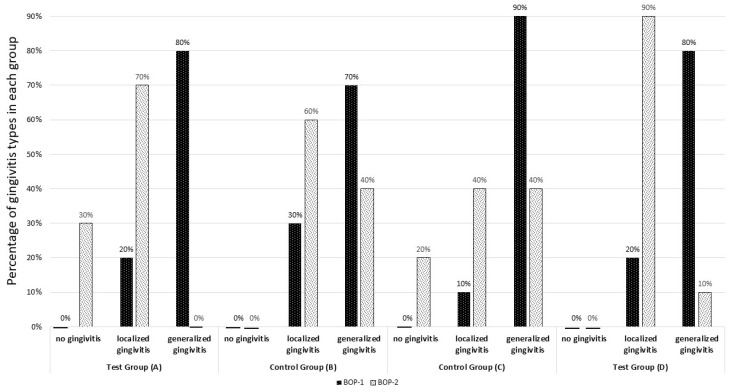
The percentage of gingivitis types in each group was determined based on a pre- and post-test assessment of bleeding on probing (BOP). BOP-1: value assessed during the first visit, BOP-2: value assessed at follow-up visit after 6 weeks of study.

The most recent edition of the Classification of Periodontal and Peri-Implant Diseases and Conditions (2017) permits the assessment of gingivitis according to its extent. Localised gingivitis is defined as 10–30% of bleeding sites, while generalised gingivitis is characterised by a prevalence of 30% or more. The results of the assessment of the patients included in this study are presented in Table 1 and Figure 4. Among all patients enrolled in the study (BOP-1), eight exhibited a localised form of gingivitis, while 32 displayed a generalised type. Following 42 days, the results were distributed as follows: five patients exhibited no signs of gingivitis, while 26 patients displayed symptoms of both the localised and generalised forms of the disease. A statistically significant change in the distribution of gingivitis types was observed in study groups A, D, and the control group C.

### 3.2. Approximal Plaque Index (API)

**Figure 5:**
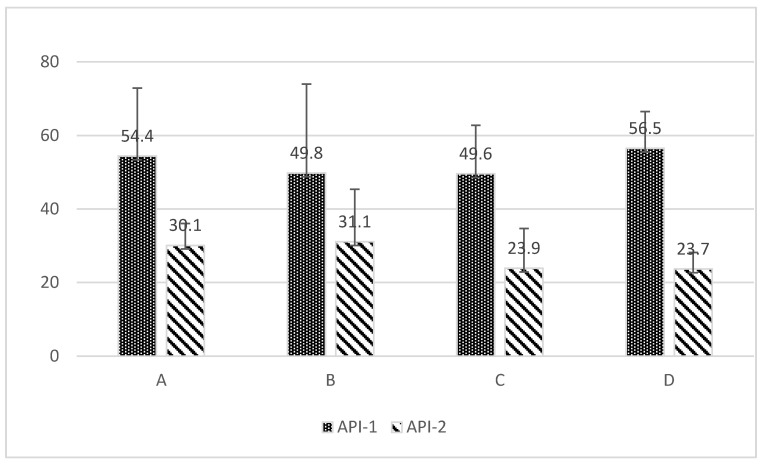
The mean percentage with standard deviation of the API index in the test groups (A and D) and control groups (B and C) is presented herewith. API-1: value assessed during the first visit. API-2: value assessed at the follow-up visit after 6 weeks of study.

Figure 5 illustrates the overall range of API distribution and mean API between groups. Patients with a baseline API between 0 and 100% were eligible to participate in the study. The participants were randomly assigned to either the study or control groups. The API value was not a criterion for inclusion or exclusion from the study. No statistically significant differences were observed between test groups A and D and control groups B and C following the pre-test (API-1: value assessed during the first visit) (A vs. B *p* = 0.556737, A vs. C *p* = 0.5398, A vs. D *p* = 0.788075, B vs. C *p* = 0.979565, B vs. D *p* = 0.393281, C vs. D *p* = 0.379458). Both the study and control groups demonstrated improvements in oral hygiene. A notable distinction in API enhancement was observed between the initial and concluding visits within the study (A, D) and control (B, C) groups (*p* = 0.012516, *p* = 0.005062, *p* = 0.004028, *p* = 0.003172, respectively). Concerning the API-2 (API-2: value assessed at the follow-up visit after 6 weeks of study) variable, statistical significance was observed for the groups in question, specifically between groups A and D (*p* = 0.014101). No statistically significant differences were observed between the remaining groups for this variable.

Furthermore, a statistically significant difference in API (API-1 vs. API-2) was confirmed for patients of all groups combined (*p* = 0.01448).

**Figure 6:**
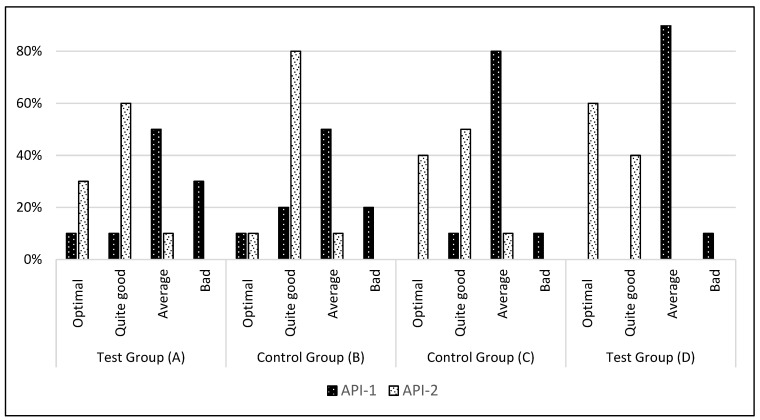
The following table presents the percentage of participants in each group who demonstrated an improvement in hygiene levels based on the pre-test and post-test API assessment. A score of 100% to 70% indicates poor hygiene, 70% to 40% indicates average hygiene, 39% to 25% indicates fairly good hygiene, and a score below 25% indicates optimal hygiene. API-1: value assessed during the first visit. API-2: value assessed at the follow-up visit after 6 weeks of study.

The Approximal Plaque Index (API) is a method of evaluating the dental hygiene of patients using a four-point scale. The four ranges are as follows: 100–70% indicates poor hygiene; 69–40% indicates average hygiene; 39–25% indicates fairly good hygiene; and less than 25% indicates optimal hygiene. The study population (API-1) exhibited a distribution of hygiene levels as follows: poor hygiene (7 patients), average hygiene (27 patients), fairly good hygiene (4 patients), and optimal hygiene (2 patients). Following a 42-day period, the results (API-2) were distributed as follows: optimal hygiene (14 patients), good hygiene (23 patients), and average hygiene (3 patients). The study results indicated that poor hygiene was present in each group of both the study and control groups during the API-1 assessment (see Table 2 and Figure 6). In the case of test group A, three patients exhibited poor hygiene, while in test group D, one patient did so. Two such patients were identified in test group B, and one patient was identified in test group C. No group, whether the test or the control, exhibited such an index value during the API-2 assessment. A comparison of the two test groups (A and D) revealed that the group that received oral hygiene instruction prior to the commencement of the experiment, which also included individual interdental cleaning instruction, exhibited lower hygiene index parameters (API-2) (*p* = 0.014101). In Group D, six patients exhibited API-2 values that met the criteria for optimal hygiene, whereas only three patients in Group A demonstrated such parameters. No statistically significant difference was observed between the API-2 results for control groups B and C (*p* = 0.218657).

### 3.3. Microbiological Investigation

The total number of microbial strains isolated from swabs taken from patients in test group A decreased from 78 microbial strains on the first day of the study to 56 strains after 42 days; in test group D, the numbers were 61 microbial strains on the first day of the study and 44 strains after 42 days of using the CBD rinse and spilanthol, respectively. In the control groups, a decline was also observed, although not to the same extent as in the test groups. The numbers of microbial strains isolated in groups B and C decreased from 58 and 61 on the first day of the study to 44 and 39 after 42 days, respectively. A decrease in the number of isolated microbial strains was observed in all groups of bacterial species observed in swabs from the test and control groups. The number of gram-positive granuloma strains isolated decreased from 35 to 28 in test group A and from 21 to 14 in test group D. In the control groups, B, the numbers were 24 cultured strains on the first day of the study and 21 on the last day, and 20 and 17 in group C, respectively. Following the application of the study rinses over a 42-day period, an increase in the number of *Streptococcus mitis* isolates from eight to ten strains and *Streptococcus salivarius* isolates from two to seven strains was observed in group A. In the second test group, D, the numbers were seven to six isolates and four to five isolates, respectively. The number of microbial strains, *including Streptococcus vestibularis* and *Streptococcus sanguinis*, remained consistent in group D. However, in group A, *S. vestibularis* exhibited a slight decrease, while *S. sanguinis* emerged as the predominant species after 42 days (Table 3A). A significant proportion of these strains constitute the physiological oral flora of patients. In the context of Gram-negative granulomas, a decline in the number of isolated strains was observed, from 19 to 15 in test group A and from 15 to 13 in test group D (Table 3B). A subsequent analysis of the results for Gram-positive bacilli yielded the following findings: in group A, a decrease from 13 to 9 strains was noted, while in group D, there was an increase from 12 to 10 strains. Among the patients in group A, there was the acquisition of *Lactobacillus casei* and *Bifidobacterium* spp., and in group D, the acquisition of the strain *Lactobacillus fermentum*, which is a group of probiotic bacteria for the human body (Table 3C). A subsequent analysis of the results for Gram-negative bacilli (which also includes periopathogens) showed a reduction in the number of strains from 11 to 4 in test group A, while in test group D, it decreased from 13 to 7. (Table 3D). Despite a statistically significant decrease in the number of strains cultured from individual patients, the species diversity within the patient groups (A and D) was maintained at the same level. The data obtained show the absence of oral sterilisation by the preparations used and the maintenance of a physiological flora, which is very important in a prophylactic measure. In the control groups, the number of isolated physiological strains was also not affected in a statistically significant manner. In addition to quantitative changes, qualitative variation in the oral microbiota was also observed, as detailed in Table 4I–IV.

## 4. Discussion

The oral cavity is home to a diverse microbiota, with its composition and activity being of particular interest to researchers. A comprehensive understanding of this microbiota is essential for evaluating its role in health and disease, as well as for assessing the impact of preventive measures and pharmaceutical interventions on its equilibrium [31,32,33]. Oral inflammation has been demonstrated to correlate with the health status of the entire organism. Prolonged, unaddressed inflammation, instigated by plaque bacteria and their interaction with the host immune system, results in the deterioration of the supporting tissues of the teeth, as well as elevated levels of inflammatory mediators, which in turn, increases the risk of developing diabetes, cardiovascular disease, and complications during pregnancy. It is also crucial to acknowledge the association between oral hygiene status and the presence of inflammation, as well as neurodegenerative diseases such as Alzheimer’s and Parkinson’s disease. It is, therefore, imperative to explore novel methods that may assist in the maintenance of oral hygiene, with a view to mitigating the likelihood of inflammation [34,35]. The primary findings of the study indicated that there was no substantial decrease or increase in the quantity of cultured bacterial strains of the physiological microbiota among any of the patient groups examined [36]. Patients in groups A and D utilised a rinse comprising TTO, spilantol, and CBD, while those in groups B and C employed TTO exclusively. For Gram-positive granulomatous microorganisms in test group A, after 42 days of rinse use, the number of cultured species decreased in a statistically non-significant manner, with a gain in *S. acidominimus* and *S. sanguinis* and an increase in the number of *S. mitis* and *S. salivarius* strains. A similar situation occurred in the second test group D, also obtaining an increase in the number of *S. salivarius* strains, while *S. sanguinis* remained at the same level. In the case of Gram-negative granulomatous microorganisms, including species of the genus *Neisseria* and *Veillonella*, no statistically significant decreases in the number of strains cultured or species diversity were observed for any of the groups assessed. It is noteworthy that both groups comprise microorganisms integral to the composition of the oral physiological flora, which reside within the oral cavity, irrespective of plaque accumulation and gingival inflammation. The application of a mouthwash comprising TTO, in conjunction with a combined mouthwash containing spilanthol and CBD, has been demonstrated to facilitate the maintenance of microbiological homeostasis within the oral cavity [32]. In test groups A and D, a decline was observed in the number of *Provotella* (A and D) and *Fusobacterium* (A) strains, which are indicative of periodontal pathogenic flora. In consideration of the intricacies inherent within the oral ecosystem, it is imperative to deliberate upon the selection of hygiene support preparations. Such preparations should facilitate the alleviation of disease symptoms, the eradication of disease-causing microorganisms, and the preservation of physiological flora. However, determining the most efficacious approach proves challenging, particularly in the case of gingivitis, where the absence of specific bacteria associated with the disease complicates the matter further. Instead, the primary focus is on the quantity and maturation of the plaque present, as well as the duration of its contact with oral surfaces. The gold standard in rinses are formulations containing chlorhexidine digluconate. However, Bescos R. et al. evaluated the effect of a 7 day rinse with 0.2% chlorhexidine on oral parameters and showed that the microbiome changed towards a higher abundance of Firmicutes and Proteobacteria species, with a lower abundance of Bacteroidetes and Fusobacteria. Concurrently, an evaluation of saliva pH revealed a shift towards an acidic milieu, a condition conducive to the increased incidence of dental caries [37].

In a separate study, Tribble et al. evaluated the impact of a different concentration of CHX gluconate mouthwash (0.12%) administered twice daily for seven days on the prevalence of bacteria colonising the tongue in healthy individuals. The study revealed that CHX led to a decline in species diversity and richness, while concurrently promoting an increase in the prevalence of Gram-negative bacteria, particularly within the Bacteroidetes (Capnocytophaga) category. This outcome stands in contrast to the findings of previous studies [38]. A definitive determination of the effect on the oral microbiota based on these studies is difficult to achieve. However, these results demonstrate that further research is required in this direction with regard to rinses with CHX, as well as a search for a replacement for its prophylactic and long-term use [38]. The present experiment demonstrated that the implementation of the mouthwash significantly improved the values of the BOP and API indices in both the test and control groups. A more pronounced distinction between the groups was observed for the BOP index, with the test groups demonstrating a higher value, which may be attributable to the anti-inflammatory effect of the supplementary ingredients present in the rinse, such as spilanthol and cannabidiol. Furthermore, it was demonstrated that groups C and D, which received hygiene training and flossing at the initial visit, exhibited a greater reduction in API. The observation that groups receiving instruction in the API assessment exhibited higher levels of reduction suggests that patient education is a crucial element in this context. Another study evaluated the impact of conducting hygiene training in a group setting on 62 patients. The change in modified OHI was assessed after 7 days and 3 and 6 months. The findings from these three follow-up studies consistently demonstrated an enhancement in the index. The index was 3.52 ± 0.7 on day 1, followed by 2.64 ± 0.69 after 7 days and 1.44 ± 0.66 after 3 months, respectively. After six months, a slight increase in the mean OHI value of 2.52 ± 0.86 was observed. These studies further underscore the pivotal role of patient education, which, however, necessitates regular repetition and consolidation to ensure optimal hygiene levels are attained [39]. Research findings indicate that a bespoke set of oral health guidelines is proving efficacious in the treatment of gingivitis and periodontitis, where adherence to oral hygiene measures is imperative for sustained improvement [40]. The utilisation of herbal medicines has been increasing on a global scale in recent years, with a growing number of individuals opting to resort to these products for the treatment of various health ailments. The increasing interest among consumers in these products can be attributed to their perceived effectiveness, natural sourcing, and affordability. This trend is evident across a wide spectrum of geographical regions, including both highly developed countries, such as those in Europe and North America, and developing countries. This surge in interest necessitates the exploration of these substances, the conducting of research, and the formulation of preparations to ensure their safety [41]. The increasing interest in cannabidiol (CBD) for oral health applications is indicative of patient preference for natural and herbal health solutions [25]. A study was conducted by Avraham et al. which demonstrated that the combination of CBD with triclosan exhibits antimicrobial activity and inhibits dental biofilm formation [42]. Barak et al. found that CBD at a concentration of 5 µg/mL reduced *S. mutans* biofilm biomass, suggesting its potential as an antitumour agent [43]. Concurrently, Paulo Peretti et al. obtained analogous results in relation to spilanthol, which also demonstrated a substantial effect on *S. mutans*. This finding suggests that spilanthol has the potential to serve as an adjuvant in dental products, aiding in the prevention and management of tooth decay. However, a direct comparison with our study is not possible at this time, as this strain was not cultured in any of the patients [22]. A number of studies have been conducted on the use of cannabidiol (CBD) in relation to saliva production. The results of these studies indicate that 58% of patients in the test group who were administered CBD lozenges reported an increase in saliva production. Saliva production is a vital component of oral health, facilitating healing, maintaining optimal pH levels, and preventing oral diseases. These products could also find use in patients with a dry mouth [26]. It is imperative to acknowledge the analgesic and anti-inflammatory properties of CBD, which, given its potential, may be utilised in various forms, such as dental gels, sprays, or rinses administered following oral procedures. Research has demonstrated that CBD can reduce the production and release of pro-inflammatory cytokines, including IL-8, IL-12, and IL-6. These are also associated with the presence of periopathogens and the presence of periodontitis. Furthermore, an increase in the levels of the anti-inflammatory cytokine IL-10 has been observed [44]. The spilanthol employed in this study has been shown to possess antibacterial, anti-inflammatory, local anaesthetic, and analgesic properties [45,46]. It has been demonstrated by other studies that the substance has the capacity to enhance saliva production. In addition, Spilanthol has demonstrated antifungal activity [45]. The combination of CBD and Spilanthol in oral products intended for the treatment of candidiasis would leverage their multifaceted properties, encompassing antimycological and analgesic effects. This approach could also address the prevalence of dry mouth, a common complaint among patients afflicted with such conditions.

A significant advantage of this study was the consistent performance of the dental examination by the same trained investigator at each visit. The VITEK^®^ MS PRIME instrument, which uses protein profile analysis for laboratory microbiological identification, is a highly accurate method. The limitations of the study can be attributed to several factors, most notably, the modest sample size. A larger sample size would have facilitated the division of the groups according to gender and age, as well as the separation of patients with significant risk factors, such as nicotine users and those with diabetes. Additionally, a larger sample size would have enabled the comparison of patients with gingivitis and patients with healthy periodontitis. A larger number of participants would have facilitated a more extensive statistical analysis. The possibility of detecting statistically significant differences between the experimental and control group was limited with a smaller sample size. In addition, a limitation of the study is the fact that the patients were not followed up outside of their time in the clinic. It is difficult to assess their behaviour, which may have a potential impact on the results obtained. However, it should be noted that the results of this study are preliminary. The paucity of information in the publications on the substances tested in dental applications, as well as their combination, limited the number of patients for the preliminary testing of the rinses prepared.

## 5. Conclusions

- The application of a mouthwash containing cannabidiol (CBD) and spilanthol has been demonstrated to maintain the homeostasis of the oral microbiome, thereby reducing the levels of bacteria that can affect periodontal health.
- The combination of TTO with spilanthol and CBD in the rinse resulted in a more significant reduction in the selected oral health parameters (BOP and API) compared to the rinse with TTO alone.
- The significant reduction in BOP index values in the test groups demonstrates the anti-inflammatory effect of the oral rinse under investigation.
- The research presented here is a pilot study, and it is important to expand the study groups and the range of concentrations of the substances in the tested rinses in future research.

## Figures and Tables

**Table 1:** BOP ranges for the test and control groups. A score of 0–9% indicates the absence of gingivitis, while a score of 10–29% indicates the presence of localised gingivitis. A score of 30–100% indicates the presence of generalised gingivitis. BOP-1: value assessed during the first visit. BOP-2: value assessed at the follow-up visit after 6 weeks of study.

	Assessment Criteria	BOP-1	BOP-2
Test group (A)	no gingivitis	-	30% (3 persons)
	localised gingivitis	20% (2 persons)	70% (7 persons)
	generalised gingivitis	80% (8 persons)	-
Control Group (B)	no gingivitis	-	-
	localised gingivitis	30% (3 persons)	60% (6 persons)
	generalised gingivitis	70% (7 persons)	40% (4 persons)
Control Group (C)	no gingivitis	-	20% (2 persons)
	localised gingivitis	10% (1 persons)	40% (4 persons)
	generalised gingivitis	90% (9 persons)	40% (4 persons)
Test group (D)	no gingivitis	-	
	localised gingivitis	20% (2 persons)	90% (9 persons)
	generalised gingivitis	80% (8 persons)	10% (1 persons)

**Table 2:** The following table presents the API index ranges for the test and control groups. A score of 100% to 70% indicates poor hygiene, 70% to 40% indicates average hygiene, 39% to 25% indicates fairly good hygiene, and a score below 25% indicates optimal hygiene. API-1: value assessed during the first visit. API-2: value assessed at the follow-up visit after 6 weeks of study.

	Assessment Criteria	API 1	API 2
Test group (A)	Optimal	10% (1 persons)	30% (3 persons)
	Quite good	10% (1 persons)	60% (6 persons)
	Average	50% (5 persons)	10% (1 persons)
	Bad	30% (3 persons)	-
Control group (B)	Optimal	10% (1 persons)	10% (1 persons)
	Quite good	20% (2 persons)	80% (8 persons)
	Average	50% (5 persons)	10% (1 persons)
	Bad	20% (2 persons)	-
Control group (C)	Optimal	-	40% (4 persons)
	Quite good	10% (1 persons)	50% (5 persons)
	Average	80% (8 persons)	10% (1 persons)
	Bad	10% (1 persons)	-
Test group (D)	Optimal	-	60% (6 persons)
	Quite good		40% (4 persons)
	Average	90% (9 persons)	-
	Bad	10% (1 persons)	-

**Table 3:** (**A**–**E**) The microbial species isolated from patients who used the mouthwash, comprising study groups (**A**,**D**) and control groups (**C**,**D**), were identified.

**A**	**A**		**B**		**C**		**D**	
	**Initial**	**Final**	**Initial**	**Final**	**Initial**	**Final**	**Initial**	**Final**
Gram (+) cocci								
*Enterococcus faecalis*	0	0	0	0	0	1	1	0
*Gemella morbillorum*	3	1	1	1	0	0	0	0
*Granulicatella elegans*	1	0	0	0	0	0	0	0
*Micrococcus luteus*	0	1	0	0	0	0	0	0
*Sarcina* spp.	0	1	1	0	0	0	0	0
*Staphylococcus aureus* MSSA	0	0	2	1	2	1	3	0
*Staphylococcus epidermidis* MSCNS	1	1	1	0	0	0	1	0
*Staphylococcus haemolyticus* MSCNS	1	1	0	0	0	0	0	0
*Staphylococcus simulans*	1	0	0	0	0	0	0	0
*Stenotrophomonas maltophilia*	0	0	0	0	1	0	0	0
*Streptococcus acidominimus*	0	1	0	1	0	1	0	0
*Streptococcus anginosus*	0	0	0	0	0	0	1	1
*Streptococcus mitis*	8	10	7	9	7	7	7	6
*Streptococcus parasanguinis*	8	0	2	2	1	1	2	0
*Streptococcus pneumoniae*	2	0	0	0	0	0	0	0
*Streptococcus pyogenes*	1	0	0	0	0	0	0	0
*Streptococcus salivarius*	2	7	4	5	6	4	4	5
*Streptococcus sanguinis*	0	1	2	1	1	1	1	1
*Streptococcus sorbinus*	1	0	0	0	0	0	0	0
*Streptococcus vestibularis*	6	4	4	1	2	1	1	1
NUMBER OF STRAINS	35	28	24	21	20	17	21	14
NUMBER OF SPECIES	12	10	9	8	7	7	9	5
**B**	**A**		**B**		**C**		**D**	
	**Initial**	**Final**	**Initial**	**Final**	**Initial**	**Final**	**Initial**	**Final**
Gram (−) cocci								
*Acidaminococcus fermentans*	0	1	0	0	0	0	0	0
*Neisseria elongata*	1	0	0	0	0	0	0	0
*Neisseria flava*	10	9	6	8	6	2	8	8
*Neisseria mucosa*	3	2	3	2	3	2	3	2
*Neisseria perflava*	0	0	2	1	0	0	0	0
*Neisseria subflava*	0	0	1	0	1	0	1	0
*Veillonella atypica*	3	1	1	0	1	1	3	2
*Veillonella parvula*	2	2	1	1	1	0	0	1
NUMBER OF STRAINS	19	15	14	12	12	5	15	13
NUMBER OF SPECIES	5	5	6	4	5	3	4	4
**C**	**A**		**B**		**C**		**D**	
	**Initial**	**Final**	**Initial**	**Final**	**Initial**	**Final**	**Initial**	**Final**
Gram (+) bacilli and rods								
*Actinomyces oris*	2	0	2	1	2	1	2	1
*Bifidobacterium breve*	0	0	0	1	0	0	0	0
*Bifidobacterium dentium*	0	2	1	0	0	0	1	0
*Bifidobacterium* spp.	0	0	0	0	0	1	0	0
*Blautia hansenii*	0	1	0	0	0	0	0	0
*Blautia producta*	0	4	1	2	0	1	0	0
*Clostridium butyricum*	1	0	0	0	0	0	0	0
*Clostridium histolyticum*	0	0	0	1	0	0	0	0
*Clostridium perfringens*	1	0	0	0	0	0	0	0
*Corynebacterium durum*	0	1	0	0	3	0	0	0
*Lactobacillus casei*	0	1	0	0	0	0	2	2
*Lactobacillus fermentum*	0	0	1	0	0	0	0	2
*Lactobacillus pentosus*	1	0	1	0	0	0	0	0
*Lactobacillus salivarius*	0	0	2	0	0	0	0	0
*Rothia aeria*	4	0	1	0	0	0	1	0
*Rothia dentocariosa*	1	0	2	1	4	1	3	4
*Rothia mucilaginosa*	3	0	1	0	3	1	3	1
*Schaalia odontolytica*	0	0	0	1	0	0	0	0
NUMBER OF STRAINS	13	9	12	7	12	5	12	10
NUMBER OF SPECIES	7	5	9	6	4	5	6	5
**D**	**A**		**B**		**C**		**D**	
	**Initial**	**Final**	**Initial**	**Final**	**Initial**	**Final**	**Initial**	**Final**
Gram (−) bacilli and rods								
*Acinetobacter baumannii*	0	0	0	0	0	1	0	0
*Bacteroides ovatus*	2	1	0	0	0	0	0	0
*Burkholderia cepacia complex*	0	0	0	0	1	0	0	0
*E. coli*	2	0	1	1	1	0	0	1
*Enterobacter cloacae*	0	0	0	0	0	1	1	0
*Fusobacterium mortiferum*	4	1	3	1	4	0	5	2
*Klebsiella aerogenes*	0	0	0	0	1	0	0	0
*Klebsiella oxytoca*	0	0	2	2	1	3	2	1
*Klebsiella pneumoniae*	0	0	1	0	2	1	0	0
*Mitsuokella multacida*	0	1	0	0	0	0	1	1
*Pantoea agglomerans*	0	0	0	0	0	1	0	1
*Prevotella denticola*	3	1	1	0	3	1	4	0
*Pseudomonas aeruginosa*	0	0	0	0	2	2	0	0
*Raoultella ornithinolytica*	0	0	0	0	1	2	0	0
*Serratia marcescens*	0	0	0	0	1	0	0	1
NUMBER OF STRAINS	11	4	8	4	17	12	13	7
NUMBER OF SPECIES	4	4	5	3	10	8	5	5
**E (summary)**	**A**		**B**		**C**		**D**	
	**Initial**	**Final**	**Initial**	**Final**	**Initial**	**Final**	**Initial**	**Final**
TOTAL NUMBER OF STRAINS	78	56	58	44	61	39	61	44
TOTAL NUMBER OF SPECIES	28	24	29	21	26	23	24	19

**Table 4:** (**I**) (I—eliminated strains) Changes in oral microbiota in patients using the mouthwash, comprising test groups A and D and control groups C and D. (**II**) (II—declined strains) Changes in oral microbiota in patients using the mouthwash, comprising test groups A and D and control groups C and D. (**III**) (III—gained strains) Changes in oral microbiota in patients using the mouthwash, comprising test groups A and D and control groups C and D. (**IV**) (IV—increased species) Changes in oral microbiota in patients using the mouthwash, comprising test groups A and D and control groups C and D.

**Changes of Microorganism Species After 42 Days of the Study**	**Test Group**		**Control Group**	
	**A**	**D**	**B**	**C**
I Eliminated strains	*Granulicatella elegans*			
	*Staphylococcus simulans*			
	*Streptococcus parasanguinis*		*Sarcina* spp.	
	*Streptococcus pneumoniae*	*Enterococcus faecalis*	*Staphylococcus epidermidis* MSCNS	*Stenotrophomonas maltophilia*
	*Streptococcus pyogenes*	*Staphylococcus aureus* MSSA	*Neisseria subflava*	*Neisseria subflava*
	*Streptococcus sorbinus*	*Staphylococcus epidermidis* MSCNS	*Veillonella atypica*	*Veillonella parvula*
	*Neisseria elongata*	*Streptococcus parasanguinis*	*Bifidobacterium dentium*	*Corynebacterium durum*
	*Actinomyces oris*	*Neisseria subflava*	*Lactobacillus fermentum*	*Burkholderia cepacia complex*
	*Clostridium butyricum*	*Bifidobacterium dentium*	*Lactobacillus pentosus*	*E. coli*
	*Clostridium perfringens*	*Rothia aeria*	*Lactobacillus salivarius*	*Fusobacterium mortiferum*
	*Lactobacillus pentosus*	*Enterobacter cloacae*	*Rothia aeria*	*Klebsiella aerogenes*
	*Rothia aeria*	*Prevotella denticola*	*Rothia mucilaginosa*	*Serratia marcescens*
	*Rothia dentocariosa*		*Klebsiella pneumoniae*	
	*Rothia mucilaginosa*		*Prevotella denticola*	
	*E. coli*			
**Changes of Microorganism Species after 42 Days of the Study**	**TEST GROUP**		**CONTROL GROUP**	
	**A**	**D**	**B**	**C**
II Declined species		*Streptococcus mitis*	*Staphylococcus aureus MSSA*	*Staphylococcus aureus MSSA*
		*Neisseria flava*	*Streptococcus sanguinis*	*Streptococcus salivarius*
	*Gemella morbillorum*	*Neisseria mucosa*	*Streptococcus vestibularis*	*Streptococcus vestibularis*
	*Streptococcus vestibularis*	*Veillonella atypica*	*Neisseria mucosa*	*Neisseria flava*
	*Neisseria flava*	*Actinomyces oris*	*Neisseria perflava*	*Neisseria mucosa*
	*Neisseria mucosa*	*Rothia mucilaginosa*	*Actinomyces oris*	*Actinomyces oris*
	*Veillonella atypica*	*Fusobacterium mortiferum*	*Rothia dentocariosa*	*Rothia dentocariosa*
	*Bacteroides ovatus*	*Klebsiella oxytoca*	*Fusobacterium mortiferum*	*Rothia mucilaginosa*
	*Fusobacterium mortiferum*			*Klebsiella pneumoniae*
	*Prevotella denticola*			*Prevotella denticola*
**Changes of Microorganism Species after 42 Days of the Study**	**TEST GROUP**		**CONTROL GROUP**	
	**A**	**D**	**B**	**C**
III Gained species	*Micrococcus luteus*			
	*Sarcina* spp.			
	*Streptococcus acidominimus*			
	*Streptococcus sanguinis*	*Veillonella parvula*	*Streptococcus acidominimus*	*Enterococcus faecalis*
	*Acidaminococcus fermentans*	*Lactobacillus fermentum*	*Bifidobacterium breve*	*Streptococcus acidominimus*
	*Bifidobacterium dentium*	*E. coli*	*Clostridium histolyticum*	*Bifidobacterium* spp.
	*Blautia hansenii*	*Pantoea agglomerans*	*Schaalia odontolytica*	*Blautia producta*
	*Blautia producta*	*Serratia marcescens*		*Acinetobacter baumannii*
	*Corynebacterium durum*			*Enterobacter cloacae*
	*Lactobacillus casei*			*Pantoea agglomerans*
	*Mitsuokella multacida*			
**Changes of Microorganism Species after 42 Days of the Study**	**TEST GROUP**		**CONTROL GROUP**	
	**A**	**D**	**B**	**C**
IV Increased species	*Streptococcus mitis*	*Streptococcus salivarius*	*Staphylococcus aureus MSSA*	*Staphylococcus aureus MSSA*
	*Streptococcus salivarius*	*Streptococcus sanguinis*	*Streptococcus mitis*	*Streptococcus mitis*
	*Veillonella parvula*	*Streptococcus vestibularis*	*Streptococcus parasanguinis*	*Streptococcus parasanguinis*
		*Lactobacillus casei*	*Streptococcus salivarius*	*Streptococcus salivarius*
		*Rothia dentocariosa*	*Neisseria flava*	*Streptococcus sanguinis*
		*Mitsuokella multacida*	*Veillonella parvula*	*Streptococcus vestibularis*
			*E. coli*	*Lactobacillus casei*
				*Rothia dentocariosa*

## Data Availability

The original contributions presented in the study are included in the article, further inquiries can be directed to the corresponding authors.

## Abbreviations

The following abbreviations are used in this manuscript:

BOP	Bleeding on probing indicator
TTO	Tea tree oil
CAL	Clinical attachment loss
CPITN	Community Periodontal Index of Treatment Needs
CBD	Cannabidiol
API	Approximal Plaque Index
TOF	Time-of-flight (TOF)
